# Cancer symptom clusters in adult patients undergoing chemotherapy: A systematic review and meta-analysis protocol

**DOI:** 10.1371/journal.pone.0273411

**Published:** 2022-09-06

**Authors:** Luís Carlos Lopes-Júnior, Taiani Ferrarini, Luciana Bicalho Cevolani Pires, Jonathan Grassi Rodrigues, Luciane Bresciani Salaroli, Karolini Zuqui Nunes

**Affiliations:** 1 Health Sciences Center at the Federal University of Espírito Santo (UFES), Vitoria, Espírito Santo, Brazil; 2 Graduate Program in Nutrition and Health at the Federal University of Espírito Santo (UFES), Vitoria, Espírito Santo, Brazil; 3 Graduate Program in Public Health at the Federal University of Espírito Santo (UFES), Vitoria, Espírito Santo, Brazil; PLOS: Public Library of Science, UNITED KINGDOM

## Abstract

**Background:**

In oncology, the prevalence of symptoms is preferentially analyzed in isolation instead of being considered in clusters. However, clinical practice shows that symptoms rarely occur separately but rather form clusters that share common underlying mechanisms in terms of intensity and severity, creating a synergistic effect among them, which can even help predict the development of future symptoms.

**Objective:**

To identify and gather evidence on the prevalence, composition, severity, and predictors of cancer symptom clusters in adult cancer patients undergoing chemotherapy.

**Methods:**

This systematic review and meta-analysis protocol was developed in compliance with PRISMA-P. Observational and experimental study designs will be included. MEDLINE/PubMed, Cochrane Library, Embase, CINAHL, PsycINFO, Web of Science, Scopus, LILACS, clinical trials.gov-NIH, the British Library, Google Scholar, and preprints [medRXiv] will be searched with no restrictions on idioms, dates, or settings. Two investigators will independently select the studies, perform data extraction, and critically appraise the risk of bias of the included studies. Heterogeneity among the studies will be assessed using the I^2^ statistic. If meta-analysis was feasible, a random-effect model analysis will be carried out. For data analysis, the pooled effect will be estimated considering 95% confidence interval and α = 5%. In addition, the certainty of evidence will be rated based on Cochrane methods in accordance with the Grading of Recommendations, Assessment, Development, and Evaluation (GRADE).

**Discussion:**

To the best of our knowledge, this systematic review and meta-analysis will be the first to identify and critically assess evidence regarding the prevalence, composition, severity, and predictors of cancer symptom clusters in adult cancer patients undergoing chemotherapy. We intend to provide health professionals with subsidies to reflect on a better understanding of symptom clusters in adult cancer patients, with the aim of contributing to the development of evidence-based therapeutic interventions and success in clinical practice.

**PROSPERO registration number:**

CRD42021248406.

## Introduction

Tumor progression and antineoplastic therapy can result in the manifestation of numerous symptoms during and after treatment, including cancer-related fatigue (CRF), sleep disturbances, pain, cognitive dysfunction, and anxiety [1, 2]. These symptoms reduce an individual’s functional status, negatively impact quality of life, and may occur alone or together, constituting clusters of symptoms [1–4]. A cluster of symptoms was initially identified in experiments with mice subjected to the induction of infectious conditions (via administration of bacterial products such as LPS11) and administration of pro-inflammatory cytokines (interleukin [IL]-1β, IL-6, tumor necrosis factor [TNF]-α) [5], exhibiting a phenomenon called sickness behavior [6, 7].

The term sickness behavior refers to a set of behavioral changes that progress with diverse infectious and inflammatory processes, for instance, the apparent loss of interest in daily activities, such as searching for food, social interaction, and sex [6]. Studies in animals and humans have shown that cytokine infusion (either systemic or central) also induces sickness behavior [8]. Similarly, in cancer patients, a phenomenon of sickness behavior is expressed, such as cancer pain, CRF, cognitive and sleep disorders, depressed mood, anxiety and depression associated with high levels of pro-inflammatory cytokines expression, including IL-1β, IL-6, IL-8, TNF-α, IL-12p70, and interferon-gamma (IFN-γ) [5–8].

There is growing consistency that common biological mechanisms may underlie the interaction between the nervous, endocrine, and immune systems, which orchestrate a set of responses capable of inducing behavioral and physiological changes in cancer patients [5–11]. In particular, studies addressing sickness behavior as well as cancer symptom clusters in cancer patients support the hypothesis that pro-inflammatory cytokines are related to the biological mechanisms underlying the emergence of these clusters [1, 5, 7, 10, 12, 13]. Evidence suggests a strong association between depression, anxiety, cachexia, and high levels of cytokines expression (IL-1β, IL-6, IL-10, TNF-α, INF-γ, and fractalkine [CX3X]) in cancer patients [14].

Studies have shown that alterations in genes encoding pro-inflammatory cytokines (IL-1β, IL-6) and their high concentrations greatly contribute to the occurrence, intensity, and severity of various symptoms in cancer patients [15, 16]. A study conducted on 599 patients recently diagnosed with lung cancer revealed that an additive effect of the mutant alleles of IL-1β, IL-10, and TNFR2 was predictive of severe pain, depressed mood, and CRF in these patients [17].

It should be noted that oncology research preferentially focuses on the prevalence of symptoms analyzed in isolation, instead of considering them as clusters. However, clinical practice shows that symptoms rarely occur separately but rather form clusters that share common underlying mechanisms in terms of intensity and severity, creating a synergistic effect among them, and can even predict the development of future symptoms [1, 2, 4, 5, 9, 10, 12, 14].

A previous systematic review of observational studies aimed to systematically assess the composition, longitudinal stability, and consistency across methodologies of common symptom clusters and their common predictors; however, it limited the search to patients with advanced cancer, focusing especially on the various statistical methods used. In addition, the authors used a generic tool for methodological assessment rather than specific tools for observational design to assess the risk of bias in the included studies [18].

Recently, a systematic review was published with the objective of evaluating the progress in symptom cluster research in adults receiving primary or adjuvant chemotherapy since 2016 and showed that psychological, gastrointestinal, and nutritional clusters were the most commonly identified clusters. Only the psychological clusters remained relatively stable over time [19].

Our review study differs in the following aspects: we did not specified a publication date or language limit (in order to minimize publication bias). Furthermore, we explored beyond observational studies by including experimental studies, and expanded the number of databases to eight, in addition to accessing gray literature and pre-prints for Health Sciences. In addition, both previous reviews used only generic tools for methodological appraisal and assessed the report of the study more than the risk of bias (in terms of internal and external validity of the studies). We used valid and design-specific tools following the recommendations of the Cochrane Collaboration [20]. Additionally, there are no systematic reviews or meta-analyses that considers a robust assessment of the risk of bias in studies involving cancer symptom clusters with validated and design-specific tools, which justifies the potential contribution of our study to the area.

This systematic review and meta-analysis aimed to identify and gather evidence on the prevalence, composition, severity, and predictors of cancer symptom clusters in adult cancer patients undergoing chemotherapy.

## Materials and methods

This systematic review and meta-analysis is in compliance with the Preferred Reporting Items for Systematic Reviews and Meta-Analyses Protocols (PRISMA-P) [21]. In addition, registration was obtained using PROSPERO/UK (registration ID: CRD42021248406).

### Search strategy

Eight electronic databases will be searched, including MEDLINE/PubMed, Cochrane Library, Embase, Web of Science, CINAHL, Scopus, PsycINFO, and LILACS. In each database, all search strategies will consider records from inception up to July 31, 2022. Additional sources will be also searched, including clinicaltrials.gov-NIH, the British Library, Google Scholar, and preprints for Health Sciences [medRXiv]. This systematic review will have no restrictions on languages or the settings of the target population. Additionally, we will scrutinize the reference lists of articles to search for additional studies [22]. The PECO acronym [23], that is, P-Population = Adult patients [≥ 18 years of age] diagnosed with malignant neoplasm; E-Exposure = Chemotherapy treatment; C-Comparison = not applicable; O-Outcomes = Prevalence, composition, stability, and severity, as well as predictors of cancer symptom clusters, was used to answer our research question (what scientific evidence is available on the prevalence, composition, severity, and predictors of cancer symptom clusters in adult cancer patients undergoing chemotherapy?).

EndNote^™^ will be used to store, organize, and manage all retrieved studies. Study selection will be conducted by two independent reviewers (LCLJ and TF) using the Rayyan^™^ application. Controlled descriptors, such as MeSH terms, Emtree terms, Thesaurus, Cinahl headings, DeCS, and their synonyms will be screened. Keywords will be also identified. The Boolean operators “AND,” “OR” and “NOT” will be employed to combine the descriptors [24, 25]. The preliminary pilot search strategy combining MeSH terms, synonyms, and keywords used in MEDLINE/PubMed is detailed in Table 1.

**Table 1:** Preliminary pilot search strategy in MEDLLINE/PubMed.

Database	Search strategy
MEDLINE/PubMed	• **Population**
	**#1**((“Young Adult” [MeSH Terms] OR “Adult” [MeSH Terms]))
	**#2** ((“Neoplasms” [MeSH Terms] OR “Neoplasia” [All Fields] OR “Neoplasias” [All Fields] OR “Neoplasm” [All Fields] OR “Tumors” [All Fields] OR “Tumor” [All Fields] OR “Cancer” [All Fields] OR “Cancers” [All Fields] OR “Malignancy” [All Fields] OR “Malignancies” [All Fields] OR “Malignant Neoplasms” [All Fields] OR “Malignant Neoplasm” [All Fields] OR “Neoplasm, Malignant” [All Fields] OR “Neoplasms, Malignant” [All Fields]))
	**# 3** #1 AND #2
	• **Exposure**
	**#4** ((“Chemotherapy” [All Fields] OR “Chemotherapy, Adjuvant” [MeSH Terms] OR “Induction Chemotherapy” [MeSH Terms] OR “Consolidation Chemotherapy” [MeSH Terms] OR “Maintenance Chemotherapy” [MeSH Terms] NOT “Radiotherapy” [MeSH Terms] NOT “Surgery” [All Fields]))
	• **Outcomes**
	**#5**((“Symptom Cluster” [All Fields] OR “Cluster, Symptom” [All Fields] OR “Clusters, Symptom” [All Fields] OR “Symptom Clusters” [All Fields] OR “Cancer Symptom Clusters” [All Fields] OR “Symptom Constellation” [All Fields] OR “Symptom Management” [All Fields]))
	**#6** #3 AND #4 AND #5

### Eligibility criteria

All observational and experimental study designs will be included.

#### Population

- Inclusion criteria: Young adults and adults of both sexes, age > 18 years, and of any ethnicity.
- Exclusion criteria: Children, adolescents, pregnant women, and elderly people with cancer of both sexes. Age in this study is defined according to the MeSH term “Aged”: a person aged 65–79 years or more.

#### Intervention/Exposure

- Inclusion criteria: Adult cancer patients undergoing chemotherapy treatment.
- Exclusion criteria: Adult cancer patients undergoing radiotherapy or oncologic surgery.

#### Outcomes

- Inclusion criteria: Prevalence, composition, severity, and predictors of cancer symptom clusters in young people and adults with cancer (> 18 years) resulting from chemotherapy treatment.
- Exclusion criteria: Studies reporting the prevalence and severity of cancer symptom clusters in young people and adults who had undergone radiotherapy or oncologic surgery.

#### Studies

- Inclusion criteria: Observational and experimental studies.
- Exclusion criteria: Qualitative studies, guidelines and reviews.

The reference lists will be searched to seek additional studies. No restrictions regarding language, period of publication, or settings will be employed.

### Study selection

**Fig 1:**
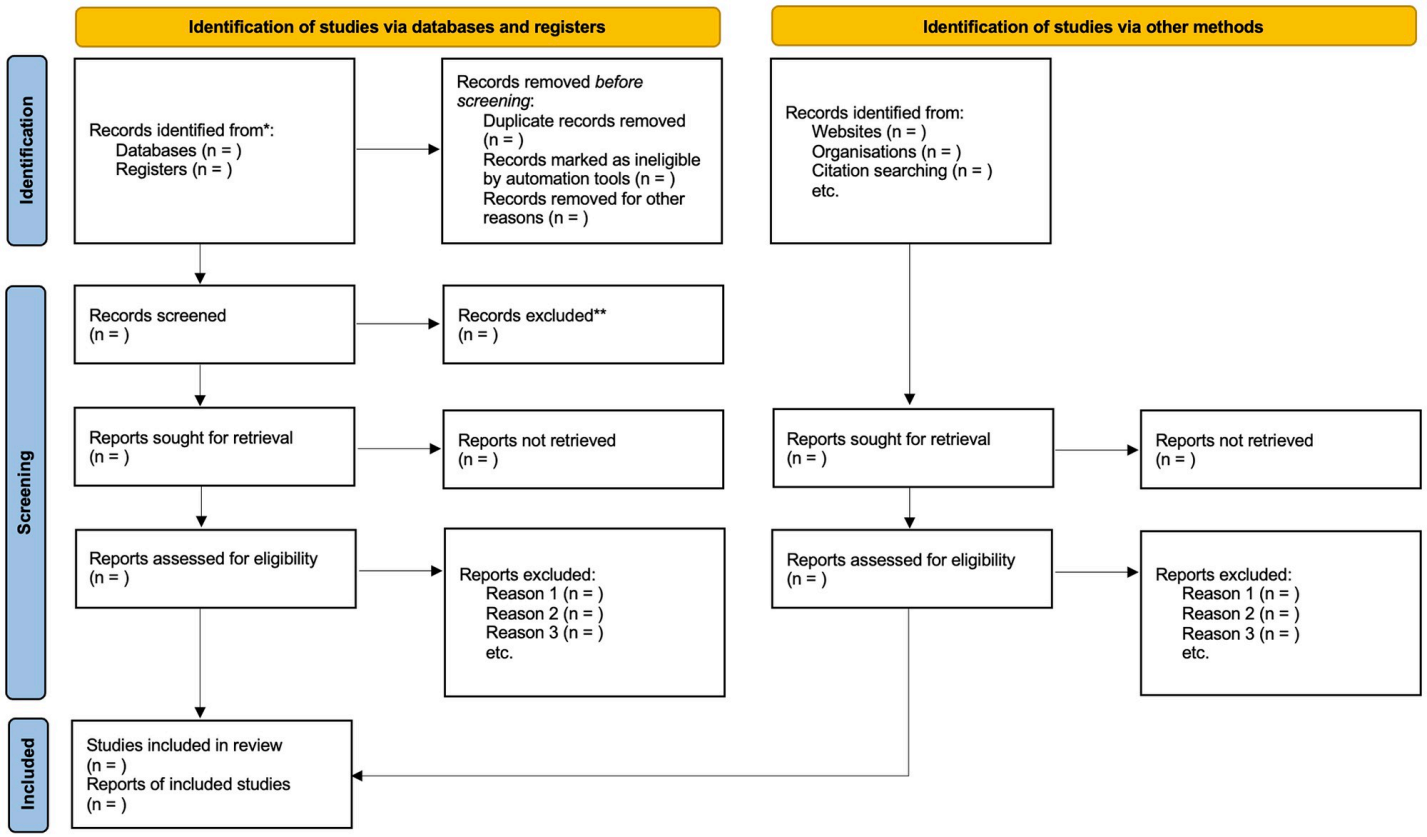
PRISMA flowchart [26].

Initially, all records scrutinized from the eight electronic databases will be imported into EndNote^™^. Thus, duplicate studies will be removed. Two independent researchers (LCLJ and TF) will be search and screen the records by titles and abstracts using the Rayyan^™^ app. Following the initial screening, the full text of the retrieved studies will be assessed for inclusion/exclusion by two independent reviewers using the Rayyan^™^ app. Disagreements in the selected studies will be resolved by a third reviewer (KZN). A flowchart summarizing the study selection process in line with the PRISMA 2020 statement [26] is presented in Fig 1.

### Data extraction and synthesis

Data extraction will be performed by two reviewers (LCLJ and TF) for each included study, based on previously published forms [22, 24, 25, 27–29]. The same two reviewers (LCLJ and TF) will independently perform the data extraction. The information to be extracted is detailed in Table 2.

**Table 2:** The data extraction form was based on previous publications [22, 24, 25, 27–29].

*Study number*:	Level of evidence:
	Methodological appraisal tool:
**STUDY CARACTERISTICS**	
Authors	
Title	
Year of publication	
Country	
Conflicts of interests	
Sponsorship	
Background	
Rationale	
Hypothesis	
Objectives	
***Methods***	
Methodology is reported is in compliance with STROBE (observational studies) or CONSORT (clinical trials)	
( ) Yes	
( ) No	
( ) Partially	
Study design	
Local:	
Sample size:	
Inclusion criteria (definition of exposure of interest)	
Exclusion criteria	
Confounding factors/Interaction factors considered	
Ethical aspects	
Procedure for data collection:	
Instruments for data collection	
Outcomes / Evaluation of outcomes (Prevalence /composition/stability/severity/ predictors of cancer symptom clusters)	
Follow-up	
Statistical analysis	
If, cohort study	I. Number of participants in the exposed and unexposed cohort:
	II. Number of participants in each group:
	III. Comparability of exposed and unexposed cohorts
	IV. Contamination (unexposed patient being exposed):
	V. Follow-up period:
	VI. Dropouts:
If, case-control study	I. Criteria for selection of cases:
	II. Criteria for selection of controls:
	III. Comparability of groups:
	IV. Dropouts:
If, experimental or quase-experimental study	**a)** Trial Register:
	**b)** Trial arms:
	• Experimental Group:
	**c)** Randomization:
	**d)** Masking:
	**e)** Intervention protocol:
	**f)** Per-protocol and modified intention-to-treat analyses:
	• Per-protocol:
	• Intention-to-treat:
	• Dropouts:
***Results***	
Main results	
Clinical significance	
Limitations of the study	
Strengths of the study	
***Conclusions***	
Main conclusions	
Implication for clinical practice and research or for decision-makers / stakeholders	

### Critical appraisal of the included studies

Initially, the level of evidence will be identified and classified according to the scale developed by the Oxford Center for Evidence-Based Medicine, which is based on the research design and classifies them as 1A, 1B, 1C, 2A, 2B, 2C, 3A, 3B, 4, and 5 [30]. The internal validity and risk of bias of randomized controlled trials will be assessed using the revised Cochrane risk-of-bias tool for randomized trials (RoB 2) [31]. In addition, to assess nonrandomized controlled trials, the risk of bias in nonrandomized studies of interventions (ROBINS-I) will be used [32]. The Newcastle-Ottawa Scale (NOS) [33] will be used to evaluate the internal validity and risk of bias of the cohort studies. The Critical Appraisal Skills Programme (CASP) tool [34] will be employed for case-control studies. Cross-sectional studies will be assessed using the Agency for Healthcare Research and Quality (AHRQ) tool [35]. The same two reviewers (LCLJ and TF) will independently perform the critical appraisal.

### Data synthesis

The study characteristics will be summarized and presented in tables. Heterogeneity among studies will be measured using the I^2^ statistic to estimate the percentage of variation across studies, ranging from 0% to 100% [36, 37], and its interpretation is as follows: a) I^2^ = 0%–40%, low heterogeneity; b) I^2^ = 30%–60%, moderate heterogeneity; c) I^2^ = 50%–90%, substantial heterogeneity; and d) I^2^ = 75%–100%, high heterogeneity [37, 38]. Based on the I^2^ statistic, we will determine whether a meta-analysis is feasible [28, 29, 39].

Moreover, subgroup analysis will be performed using a random-effect model adjusted for age, sex, cancer symptom cluster prevalence, and cancer type. For data analysis, the pooled effect estimates will be calculated considering a 95% confidence interval (CI) and alpha set at 0.05, using R statistical software v. 4.0.4 (R Foundation for Statistical Computing, Vienna, Austria). In addition, the certainty of evidence will be rated based on Cochrane methods and in accordance with the Grading of Recommendations, Assessment, Development, and Evaluation (GRADE) [40]. Two reviewers (LCLJ and LBCP) will independently perform the critical appraisal of the evidence to assess the studies. Disagreements will be resolved by a third reviewer (KZN).

### Ethics aspects and plans for dissemination

Ethical approval was not required for this study design. Moreover, this systematic review and meta-analysis will be conducted following the PRISMA 2020 statement [26]. Regarding plans for dissemination, we intend to disclose the results via peer-reviewed publications and presentations at international conferences.

## Discussion

Indeed, there is a high prevalence of cancer symptom clusters in adult cancer patients, and such unpleasant symptoms are reported daily by patients and health professionals [4, 9, 16] in clinical practice. These symptom clusters are associated with worse prognosis and decreased quality of life. Thus, its effective management is crucial in oncology. The importance of evaluating and intervening in cancer symptom clusters, not only in terms of patient survival rate but also in terms of quality of life across the treatment, is a priority and an integral part of the pillars of research in oncology and for the advancement of science in symptom management in oncology [41–45].

## Conclusion

To the best of our knowledge, this systematic review will be the first to identify and critically assess evidence of the prevalence, composition, severity, and predictors of cancer symptom clusters in adult cancer patients undergoing chemotherapy. We intend to provide health professionals with subsidies to reflect on a better understanding of symptom clusters in adult cancer patients undergoing chemotherapy, with the aim of contributing to the development of evidence-based therapeutic strategies and success in clinical practice.

## Supporting information

S1 Checklist PRISMA checklist. (DOCX)

## References

[1] Kim HJ, Barsevick AM, Fang CY, Miaskowski C. Common biological pathways underlying the psychoneurological symptom cluster in cancer patients. Cancer Nurs. 2012 Nov-Dec;35(6):E1–E20. doi: 10.1097/NCC.0b013e318233a811 . 22228391

[2] Dodd MJ, Miaskowski C, Lee KA. Occurrence of symptom clusters. J Natl Cancer Inst Monogr. 2004;(32):76–8. doi: 10.1093/jncimonographs/lgh008 . 15263044

[3] Amorim MH, Lopes-Jünior LC. Psychoneuroimmunology and Nursing research: discovery, paradigm shifts, and methodological innovations. Acta Paul Enferm. 2021;34:e-EDT1. 10.37689/acta-ape/2021EDT1

[4] Lopes-Júnior LC, Olson K, de Omena Bomfim E, Pereira-da-Silva G, Nascimento LC, de Lima RA. Translational research and symptom management in oncology nursing. Br J Nurs. 2016 May 26-Jun 8;25(10):S12, S14, S16 passim. doi: 10.12968/bjon.2016.25.10.S12 . 27231745

[5] Barsevick AM, Whitmer K, Nail LM, Beck SL, Dudley WN. Symptom cluster research: conceptual, design, measurement, and analysis issues. J Pain Symptom Manage. 2006 Jan;31(1):85–95. doi: 10.1016/j.jpainsymman.2005.05.015 . 16442485

[6] Kent S, Bluthé RM, Kelley KW, Dantzer R. Sickness behavior as a new target for drug development. Trends Pharmacol Sci. 1992 Jan;13(1):24–8. doi: 10.1016/0165-6147(92)90012-u . 1542935

[7] Dantzer R, Kelley KW. Twenty years of research on cytokine-induced sickness behavior. Brain Behav Immun. 2007 Feb;21(2):153–60. doi: 10.1016/j.bbi.2006.09.006 . 17088043 PMC1850954

[8] Ader R. On the development of psychoneuroimmunology. Eur J Pharmacol. 2000 Sep 29;405(1–3):167–76. doi: 10.1016/s0014-2999(00)00550-1 . 11033324

[9] Lopes-Júnior LC, Tuma MC, Amorim MHC. Psychoneuroimmunology and the Oncology Nursing: theoretical essay. Rev Esc Enf USP. 2021;10;55:e20210159. doi: 10.1590/1980-220X-REEUSP-2021-0159 34516609

[10] Green McDonald P, O’Connell M, Lutgendorf SK. Psychoneuroimmunology and cancer: a decade of discovery, paradigm shifts, and methodological innovations. Brain Behav Immun. 2013 Mar;30 Suppl(0):S1–9. Epub 2013 Jan 16. doi: 10.1016/j.bbi.2013.01.003 . 23333846 PMC3907949

[11] Lopes-Júnior LC, Urbano IR, Schuab SIPC, Pessanha RM, Rosa GS, Lima RAG. Effectiveness of complementary therapies for the management of symptom clusters in palliative care in pediatric oncology: a systematic review. Rev Esc Enferm USP. 2021 May 19;55:03709. doi: 10.1590/S1980-220X2020025103709 . 34037196

[12] Dantzer R, Meagher MW, Cleeland CS. Translational approaches to treatment-induced symptoms in cancer patients. Nat Rev Clin Oncol. 2012 May 29;9(7):414–26. doi: 10.1038/nrclinonc.2012.88 . 22641361 PMC3412618

[13] Lopes-Júnior LC, Pereira-da-Silva G, Silveira DSC, Veronez LC, Santos JC, Alonso JB, et al. The Effect of Clown Intervention on Self-Report and Biomarker Measures of Stress and Fatigue in Pediatric Osteosarcoma Inpatients: A Pilot Study. Integr Cancer Ther. 2018 Sep;17(3):928–940. Epub 2018 Jun 14. doi: 10.1177/1534735418781725 . 29900751 PMC6142113

[14] Seruga B, Zhang H, Bernstein LJ, Tannock IF. Cytokines and their relationship to the symptoms and outcome of cancer. Nat Rev Cancer. 2008 Nov;8(11):887–99. Epub 2008 Oct 10. doi: 10.1038/nrc2507 . 18846100

[15] Bower JE, Ganz PA, Tao ML, Hu W, Belin TR, Sepah S, et al. Inflammatory biomarkers and fatigue during radiation therapy for breast and prostate cancer. Clin Cancer Res. 2009 Sep 1;15(17):5534–40. Epub 2009 Aug 25. doi: 10.1158/1078-0432.CCR-08-2584 . 19706826 PMC2884979

[16] Miaskowski C, Dodd M, Lee K, West C, Paul SM, Cooper BA, et al. Preliminary evidence of an association between a functional interleukin-6 polymorphism and fatigue and sleep disturbance in oncology patients and their family caregivers. J Pain Symptom Manage. 2010 Oct;40(4):531–44. Epub 2010 Jun 8. doi: 10.1016/j.jpainsymman.2009.12.006 . 20570482 PMC2952712

[17] Reyes-Gibby CC, Wang J, Spitz M, Wu X, Yennurajalingam S, Shete S. Genetic variations in interleukin-8 and interleukin-10 are associated with pain, depressed mood, and fatigue in lung cancer patients. J Pain Symptom Manage. 2013 Aug;46(2):161–72. Epub 2012 Nov 11. doi: 10.1016/j.jpainsymman.2012.07.019 . 23149083 PMC3578112

[18] Dong ST, Butow PN, Costa DS, Lovell MR, Agar M. Symptom clusters in patients with advanced cancer: a systematic review of observational studies. J Pain Symptom Manage. 2014 Sep;48(3):411–50. doi: 10.1016/j.jpainsymman.2013.10.027 24703941

[19] Harris CS, Kober KM, Conley YP, Dhruva AA, Hammer MJ, Miaskowski CA. Symptom clusters in patients receiving chemotherapy: A systematic review. BMJ Support Palliat Care. 2022 Mar;12(1):10–21. doi: 10.1136/bmjspcare-2021-003325 34921000 PMC8857036

[20] Higgins JPT, Thomas J, Chandler J, Cumpston M, Li T, Page MJ, editors. Cochrane Handbook for Systematic Reviews of Interventions version 6.2 (updated February 2021). Cochrane, 2021. www.training.cochrane.org/handbook

[21] Shamseer L, Moher D, Clarke M, Ghersi D, Liberati A, Petticrew M, et al. Preferred reporting items for systematic review and meta-analysis protocols (PRISMA-P) 2015: elaboration and explanation. BMJ. 2015 Jan 2;350:g7647. doi: 10.1136/bmj.g7647 . 25555855

[22] Lopes-Júnior LC, Bomfim E, Olson K, Neves ET, Silveira DSC, Nunes MDR, et al. Effectiveness of hospital clowns for symptom management in paediatrics: systematic review of randomised and non-randomised controlled trials. BMJ. 2020 Dec 16;371:m4290. doi: 10.1136/bmj.m4290 . 33328164 PMC7737653

[23] Morgan RL, Whaley P, Thayer KA, Schünemann HJ. Identifying the PECO: A framework for formulating good questions to explore the association of environmental and other exposures with health outcomes. Environ Int. 2018 Dec;121(Pt 1):1027–1031. Epub 2018 Aug 27. doi: 10.1016/j.envint.2018.07.015 . 30166065 PMC6908441

[24] Lopes-Júnior LC, Rosa GS, Pessanha RM, Schuab SIPC, Nunes KZ, Amorim MHC. Efficacy of the complementary therapies in the management of cancer pain in palliative care: A systematic review. Rev Lat Am Enfermagem. 2020 Sep 30;28:e3377. doi: 10.1590/1518-8345.4213.3377 . 33027406 PMC7529450

[25] Lopes-Júnior LC, Rosa MADRP, Lima RAG. Psychological and Psychiatric Outcomes Following PICU Admission: A Systematic Review of Cohort Studies. Pediatr Crit Care Med. 2018 Jan;19(1):e58–e67. doi: 10.1097/PCC.0000000000001390 . 29189670

[26] Page MJ, McKenzie JE, Bossuyt PM, Boutron I, Hoffmann TC, Mulrow CD, et al. The PRISMA 2020 statement: an updated guideline for reporting systematic reviews. BMJ 2021 Mar 29;372(71). doi: 10.1136/bmj.n71 . 33782057 PMC8005924

[27] Gonçalves CA, Lopes-Júnior LC, Nampo FK, Zilly A, Mayer PCM, Pereira-da-Silva G. Safety, efficacy and immunogenicity of therapeutic vaccines in the treatment of patients with high-grade cervical intraepithelial neoplasia associated with human papillomavirus: a systematic review protocol. BMJ Open 2019 Jul 17;9(7):e026975. doi: 10.1136/bmjopen-2018-026975 . 31320349 PMC6661674

[28] Lopes-Júnior LC, Bomfim E, Silveira DSCD, Pessanha RM, Schuab SIPC, Lima RAG. Effectiveness of mass testing for control of COVID-19: a systematic review protocol. BMJ Open. 2020;10(8):e040413. doi: 10.1136/bmjopen-2020-040413 . 32847926 PMC7451293

[29] Silva FJGD Junior, Sales JCES, Monteiro CFS, Costa APC, Campos LRB, Miranda PIG, et al. Impact of COVID-19 pandemic on mental health of young people and adults: a systematic review protocol of observational studies. BMJ Open. 2020 Jul 1;10(7):e039426. doi: 10.1136/bmjopen-2020-039426 . 32611746 PMC7358102

[30] Oxford Centre for Evidence-Based Medicine. Levels of Evidence Working Group. "The Oxford 2011 Levels of Evidence". Oxford Centre for Evidence-Based Medicine. 2011.

[31] Sterne JAC, Savović J, Page MJ, Elbers RG, Blencowe NS, Boutron I, et al. RoB 2: a revised tool for assessing risk of bias in randomised trials. BMJ. 2019 Aug 28;366:l4898. doi: 10.1136/bmj.l4898 . 31462531

[32] Sterne JA, Hernán MA, Reeves BC, Savović J, Berkman ND, Viswanathan M, et al. ROBINS-I: a tool for assessing risk of bias in non-randomised studies of interventions. BMJ. 2016 Oct 12;355:i4919. doi: 10.1136/bmj.i4919 . 27733354 PMC5062054

[33] Wells GA, Shea B, O’Connell D, Peterson J, Welch V, Losos M, et al. The Newcastle-Ottawa Scale (NOS) for assessing the quality if nonrandomized studies in meta-analyses. http://www.ohri.ca/programs/clinical_epidemiology/oxford.htm

[34] Critical Appraisal Skills Programme. CASP (Case Control Study) Checklist [online] https://casp-uk.net/casp-tools-checklists/. Accessed: Date Accessed in July 24, 2021.

[35] Methods Guide for Effectiveness and Comparative Effectiveness Reviews [Internet]. Rockville (MD): Agency for Healthcare Research and Quality (US); 2008. 21433403

[36] Higgins JPT, Thompson SG. Quantifying heterogeneity in a meta-analysis. Stat Med jun 2002; 21(11): 1539–1558. doi: 10.1002/sim.1186 . 12111919

[37] Higgins JPT, Thompson SG, Deeks JJ, Altman DG. Measuring inconsistency in metaanalyses. BMJ sep 2003; 327(7414): 557–60. doi: 10.1136/bmj.327.7414.557 . 12958120 PMC192859

[38] Fokoua-Maxime CD, Lontchi-Yimagou E, Cheuffa-Karel TE, Tchato-Yann TL, Pierre-Choukem S. Prev- alence of asymptomatic or "silent" myocardial ischemia in diabetic patients: Protocol for a systematic review and meta-analysis. PLoS One 2021 Jun 10; 16(6):e0252511. doi: 10.1371/journal.pone.0252511 . 34111136 PMC8191872

[39] Lopes-Júnior LC, Siqueira PC, Maciel ELN. School reopening and risks accelerating the COVID-19 pandemic: A systematic review and meta-analysis protocol. PLoS One. 2021 Nov 17;16(11):e0260189. doi: 10.1371/journal.pone.0260189 34788344 PMC8598030

[40] Balshem H, Helfand M, Schünemann HJ, et al. GRADE guidelines: 3. Rating the quality of evidence. J Clin Epidemiol 2011; 64: 401–406. doi: 10.1016/j.jclinepi.2010.07.015 . 21208779

[41] Miaskowski C, Barsevick A, Berger A, Casagrande R, Grady PA, Jacobsen P, et al. Advancing Symptom Science Through Symptom Cluster Research: Expert Panel Proceedings and Recommendations. J Natl Cancer Inst. 2017 Jan 24;109(4):djw253. doi: 10.1093/jnci/djw253 . 28119347 PMC5939621

[42] Lopes-Júnior LC, Veronez LC. Circadian rhythms disruption in cancer. *Biol Rhythm Res*, 2022;53(9):1382–1399. doi: 10.1080/09291016.2021.1951470

[43] Lopes-Júnior LC. Personalized Nursing Care in Precision-Medicine Era. SAGE Open Nurs. 2021 Dec 9;7:23779608211064713. doi: 10.1177/23779608211064713 35174279 PMC8841427

[44] Lopes Júnior LC. The era of precision medicine and its impact on nursing: paradigm shifts? Rev Bras Enferm. 2021 Jul 28;74(5):e740501. doi: 10.1590/0034-7167.2021740501 34346956

[45] Von Ah D, Brown CG, Brown SJ, Bryant AL, Davies M, Dodd M, et al. Research Agenda of the Oncology Nursing Society: 2019–2022. Oncol Nurs Forum. 2019 Nov 1;46(6):654–669. doi: 10.1188/19.ONF.654-669 . 31626621

