# Supplementary material for: Cancer symptom clusters in adult patients undergoing chemotherapy: A systematic review and meta-analysis protocol
Source: PLoS One. 2022 Sep 6;17(9):e0273411. doi: 10.1371/journal.pone.0273411 (PMC9447873; doi:10.1371/journal.pone.0273411)
Supplement: Attachment — Submitted filename: Response to Reviewers .docx [file pone.0273411.s002.docx]

**Response to Reviewers**

PONE-D-21-25793

Cancer symptom clusters in adult patients undergoing chemotherapy: a systematic review protocol

Dear Dr. Lopes-Junior,

Thank you for submitting your manuscript to PLOS ONE. After careful consideration, we feel that it has merit but does not fully meet PLOS ONE’s publication criteria as it currently stands. Therefore, we invite you to submit a revised version of the manuscript that addresses the points raised during the review process.
We have now received reports from our referees of your manuscript, as agree with reviewers comments raised a few concerns about this study. After careful consideration, we invite you to submit a revised version of the manuscript.  
Please submit your revised manuscript by Jun 20 2022 11:59PM. If you will need more time than this to complete your revisions, please reply to this message or contact the journal office at [plosone@plos.org](mailto:plosone@plos.org). When you're ready to submit your revision, log on to [https://www.editorialmanager.com/pone/](https://www.editorialmanager.com/pone/) and select the 'Submissions Needing Revision' folder to locate your manuscript file.

Please include the following items when submitting your revised manuscript:

- A rebuttal letter that responds to each point raised by the academic editor and reviewer(s). You should upload this letter as a separate file labeled 'Response to Reviewers'.
- A marked-up copy of your manuscript that highlights changes made to the original version. You should upload this as a separate file labeled 'Revised Manuscript with Track Changes'.
- An unmarked version of your revised paper without tracked changes. You should upload this as a separate file labeled 'Manuscript'.

We look forward to receiving your revised manuscript.

Kind regards,

Senthilnathan Palaniyandi, Ph.D
Academic Editor
PLOS ONE

Journal requirements:

When submitting your revision, we need you to address these additional requirements.

1. Please ensure that your manuscript meets PLOS ONE's style requirements, including those for file naming. The PLOS ONE style templates can be found at

<https://journals.plos.org/plosone/s/file?id=wjVg/PLOSOne_formatting_sample_main_body.pdf> and

<https://journals.plos.org/plosone/s/file?id=ba62/PLOSOne_formatting_sample_title_authors_affiliations.pdf>.

[Note: HTML markup is below. Please do not edit.]

Reviewers' comments:

Reviewer's Responses to Questions

**Comments to the Author**

1. Does the manuscript provide a valid rationale for the proposed study, with clearly identified and justified research questions?

The research question outlined is expected to address a valid academic problem or topic and contribute to the base of knowledge in the field.

Reviewer #1: Yes

Reviewer #2: No

Reviewer #3: Yes

2. Is the protocol technically sound and planned in a manner that will lead to a meaningful outcome and allow testing the stated hypotheses?

The manuscript should describe the methods in sufficient detail to prevent undisclosed flexibility in the experimental procedure or analysis pipeline, including sufficient outcome-neutral conditions (e.g. necessary controls, absence of floor or ceiling effects) to test the proposed hypotheses and a statistical power analysis where applicable. As there may be aspects of the methodology and analysis which can only be refined once the work is undertaken, authors should outline potential assumptions and explicitly describe what aspects of the proposed analyses, if any, are exploratory.

Reviewer #1: Yes

Reviewer #2: Partly

Reviewer #3: Yes

3. Is the methodology feasible and described in sufficient detail to allow the work to be replicable?


Descriptions of methods and materials in the protocol should be reported in sufficient detail for another researcher to reproduce all experiments and analyses. The protocol should describe the appropriate controls, sample size calculations, and replication needed to ensure that the data are robust and reproducible.

Reviewer #1: Yes

Reviewer #2: Yes

Reviewer #3: Yes

4. Have the authors described where all data underlying the findings will be made available when the study is complete?

The [PLOS Data policy](https://journals.plos.org/plosone/s/materials-and-software-sharing) requires authors to make all data underlying the findings described in their manuscript fully available without restriction, with rare exception, at the time of publication. The data should be provided as part of the manuscript or its supporting information, or deposited to a public repository. For example, in addition to summary statistics, the data points behind means, medians and variance measures should be available. If there are restrictions on publicly sharing data—e.g. participant privacy or use of data from a third party—those must be specified.

Reviewer #1: Yes

Reviewer #2: Yes

Reviewer #3: Yes

5. Is the manuscript presented in an intelligible fashion and written in standard English?

PLOS ONE does not copyedit accepted manuscripts, so the language in submitted articles must be clear, correct, and unambiguous. Any typographical or grammatical errors should be corrected at revision, so please note any specific errors here.

Reviewer #1: Yes

Reviewer #2: No

Reviewer #3: Yes

**6. Review Comments to the Author**

**Reviewer #1:** I appreciate the opportunity to review this article protocol. This  systematic review is important in clinical practice, especially in  the developing countries that have high mortality rates in cancer patients.There are currently no comments on the study protocol, but there is the systematic review (Skye Tian Dong, et al 2014 "Symptom Clusters in Patients With Advanced Cancer: A Systematic Review of  Observational Studies"), I hope to return to. I wish the researchers success and await such the important  systematic review. No need to chart1

**Response:** Thank you so much for your comments! We have added a paragraph mentioning this review (Dong et al., 2014) in the introduction and specified how ours differs from this one as well as the current one by Harris et al., 2022. In addition, we have removed the chart 1 as per suggested. Thanks!

**Reviewer #2:** The authors aim to identify and gather evidence on the prevalence, composition, severity, and predictors of cancer symptom clusters in adult cancer patients undergoing chemotherapy. However, a similar study has recently been carried out elsewhere and the results have been published: Harris CS, Kober KM, Conley YP, et alSymptom clusters in patients receiving chemotherapy: A systematic review; BMJ Supportive & Palliative Care 2022;12:10-21. Hence, this study is not warranted.

**Response:** We have added a paragraph mentioning this review (Dong et al., 2014) in the introduction and specified how ours differs from this one.

“Recently, a systematic review was published with the objective of evaluating the progress in symptom clusters research in adults receiving primary or adjuvant chemotherapy since 2016, and showed that psychological, gastrointestinal, and nutritional clusters were the most commonly identified clusters. Only the psychological clusters remained relatively stable over time [19].

It is stand out that our review study differs from the latter in the following aspects: we will not specify a publication date or language limit (in order to minimize publication bias). Furthermore, we will explore beyond observational studies, as we will include experimental studies and also, we will expand the number of databases to 8, besides the access gray literature and pre-prints for Health Sciences. Another difference is that both previous reviews used only generic tools for methodological appraisal and that assess the report of the study more than the risk of bias (in terms of internal validity and external validity of the studies). Here, we will use valid and design-specific tools, following the recommendations of the Cochrane Collaboration [20]. Additionally, there is still no systematic review and metanalysis taking into account a robust assessment of the risk of bias in studies involving cancer symptom clusters with validated and design-specific tools, which justifies our study for a potential contribution to the area”.

**Reviewer #3:** The present is a protocol aiming to capture clusters of symptoms of patients undergoing chemiotherapy. The paper is very clear regarding goal source of participants and outcomes

**Response:** Thank you so much!

Some issues are needed

1) Abstract and full paper>why will search be stopped on August 2021?

**Response:** In fact, we put August, 2021 because that was the date when we submitted the article to PLOS ONE. However, we will update the search once the protocol is accepted. We edit this in the paper. Thank you!

2) Introduction and methods>authors spoke correctly about physical and physiological symptoms. Do they think subgroup analysis may be needed?

**Response:** This is an important point to consider. Thank you very much! Yes, probably depending on the results we will find we will do subgroup analysis. We really appreciated this comment. I have added one paragraph on the sub-item “Data synthesis” into the methods section.

3) Methods: elderly patients should be defined with a clear cut off or reported according to definition of each papers

**Response:** Thanks for this comment. We have added one sentence in order to make it clearer. “Aged in this study is defined according to MeSH term “Aged”: a person 65 through 79 years of age or more”.

[**Aged**](https://www.ncbi.nlm.nih.gov/mesh/68000368)

A person 65 through 79 years of age. For a person older than 79 years, AGED, 80 AND OVER is available.

Year introduced: 1966

4) Methods: regarding features of the studies I’m not sure that impact factor may be so relevant (just a comments) as IF does not always relate with quality of the paper

**Response:** Indeed, we agreed with you. So, we have removed. Thanks!

5) Conflict of interests>please define better

**Response:** The Committee on Publication Ethics (COPE) states in its Guidelines on Good Publication Practice (2003) that: 'Conflicts of interest arise when authors, reviewers, or editors have interests that are not fully apparent and that may influence their judgments on what is published.

6) Methods>do authors think that a subgroup analysis for kind of cancer and for protocol of chemiptherapy may be useful?

**Response:** This point is very interesting. Studies with cancer symptom clusters are quite heterogeneous around the target population, types of cancers included in the analyses, tumor staging and chemotherapy protocol. Thus, this stratification can be carried out depending on the evidence that we will find, especially if the meta-analysis is feasible. I have added this on “Data Synthesis”. Many Thanks!

7) Moreover, do they think that some quantitative analysis (e.g meta-regression to evaluate impact of gender, age,) on clusters may be of interest?

**Response:** Yes. I specified this in the topic “Data Syjthesis”. Thank you so much for your valuable contribuitions.

8) Methods: primary and secondary end points should be better defined

**Response:** We have edited this in the chart 1. Actually, our outcomes are including: “Prevalence /composition/stability/ severity/ predictors of cancer symptom clusters, as stated in the PECO acronym.

7. PLOS authors have the option to publish the peer review history of their article ([what does this mean?](https://journals.plos.org/plosone/s/editorial-and-peer-review-process#loc-peer-review-history)). If published, this will include your full peer review and any attached files.


If you choose “no”, your identity will remain anonymous but your review may still be made public.


**Do you want your identity to be public for this peer review?** For information about this choice, including consent withdrawal, please see our [Privacy Policy](https://www.plos.org/privacy-policy).

Reviewer #1: No

Reviewer #2: No

Reviewer #3: **Yes:** Fabrizio D'Ascenzo
