# Supplementary material for: Cancer symptom clusters in adult patients undergoing chemotherapy: A systematic review and meta-analysis protocol
Source: PLoS One. 2022 Sep 6;17(9):e0273411. doi: 10.1371/journal.pone.0273411 (PMC9447873; doi:10.1371/journal.pone.0273411)
Supplement: Attachment — Submitted filename: Response to Reviewers.docx [file pone.0273411.s003.docx]

**Response to Reviewers**

Vitória, ES, Brazil, July 15th, 2022

***Minor revision***

PONE-D-21-25793R1

**Cancer symptom clusters in adult patients undergoing chemotherapy: a systematic review and metanalysis protocol**

Dear Dr. Lopes-Júnior,

Thank you for submitting your manuscript to PLOS ONE. After careful consideration, we feel that it has merit but does not fully meet PLOS ONE’s publication criteria as it currently stands. Therefore, we invite you to submit a revised version of the manuscript that addresses the points raised during the review process.

Your manuscript has been reassessed by the three reviewers from the previous round, whose reports can be found below. As you will see from the comments, the reviewers acknowledge that the manuscript has improved significantly.

**Response:** Dear Dr. Joseph Donlan,

Thank you for the opportunity to review the manuscript after the reviewers' suggestions and recommendations.

All points were addressed and/or clarified in this new version. In addition, we responded item by item to the questions raised by the reviewers in this letter.

While assessing your manuscript, we noticed several errors in the use of spelling, punctuation and grammar throughout the manuscript. So that your manuscript meets our publication criterion requiring that 'the article is presented in an intelligible fashion and is written in standard English.', we request that you thoroughly copyedit your manuscript to fix these errors. You may wish to use a professional scientific editing service to do this.

**Response:** Ok. As suggested, we have done it. So, we have paid an English proofreading to Editage (<https://www.editage.com>) for polishing the text and correct grammar and spelling mistakes according to the cultured language. Therefore, an extensive review of English was carried out.

We look forward to receiving your revised manuscript.

Kind regards,

Joseph Donlan
Editorial Office
PLOS ONE

**Journal Requirements:**

Please review your reference list to ensure that it is complete and correct. If you have cited papers that have been retracted, please include the rationale for doing so in the manuscript text, or remove these references and replace them with relevant current references. Any changes to the reference list should be mentioned in the rebuttal letter that accompanies your revised manuscript. If you need to cite a retracted article, indicate the article’s retracted status in the References list and also include a citation and full reference for the retraction notice.
**Response:** We have carefully reviewed the reference list and we ensure that it is complete and correct. Also, we have no papers retracted cited in this manuscript.

**Review Comments to the Author**

Reviewer #1: When mentioning this review(...2014), this review was not intended in itself, but rather the intent was, the authors should clarify the strengths of their study and its difference from what preceded it, especially since the authors indicated in the study protocol that (this systematic review and comprehensive analysis will be the first to identify and evaluate Evidence about prevalence, composition, and severity ....)
I wish you success in publication a valuable study.

**Response:** OK. We addressed this in the introduction. Thank you so much!

Reviewer #2: Thank you for addressing the earlier comments. The manuscript needs a thorough proof reading for syntactic errors and grammars, especially in the paragraphs that have been added now to address the reviewers' comments.

**Response:** Thank you so much! We have paid an English proofreading to Editage (https://www.editage.com) for polishing the text and correct grammar and spelling mistakes according to the cultured language. Therefore, an extensive review of English was carried out.

Reviewer #3: all comments have been addressed.
i have no further comments, because the authors cleary detailed all the comments

**Response:** Thank you so much!

Reviewer #1: **Yes:** Rania E Moustafa

Reviewer #2: No

Reviewer #3: **Yes:** Fabrizio D'Ascenzo
